# A case report of cardia carcinoma: Underwent Heller myotomy for Achalasia

**DOI:** 10.1097/MD.0000000000036924

**Published:** 2024-01-26

**Authors:** Lin-qi Wen, Da-wei Wei

**Affiliations:** a Thoracic Surgery Department, HePing Hospital Affilated to Changzhi Medical College, Changzhi City, Shanxi Province, China.

**Keywords:** achalasia, gastric cardia adenocarcinoma, Heller myotomy, case report

## Abstract

**Background:**

One of the risk factors for esophageal adenocarcinoma is achalasia, an esophageal motility disorder that is typically treated surgically through laparotomy or laparoscopic surgery. The risk factors of gastric cardia cancer are also similar to esophageal adenocarcinoma due to the anatomical location of the gastric cardia close to the esophagus. There is currently no clinical evidence that achalching has a correlation with gastric cardia cancer.

**Case summary:**

We report the case of an 85-year-old female patient was admitted to our department with dysphagia for 6 months. She underwent a dissecting Heller myotomy for pancreatic achalasia in 2006, with occasional postoperative symptoms of reflux and heartburn. Outpatient upper gastrointestinal imaging was suggestive of cardia cancer, and gastroscopic pathological findings were suggestive of moderately-lowly-differentiated adenocarcinoma. The patient was admitted to the operating room on August 30, 2022 to undergo radical pancreatic cancer surgery plus abdominal adhesion release, and postoperative review of the upper gastrointestinal imaging showed a patent anastomosis with no spillage, filling of the residual stomach, and duodenal visualization.

**Conclusion:**

Postoperative patients with achalasia often have symptoms of reflux, which may be one of the factors for the development of pancreatic cancer in this patient, thus requiring clinicians to pay more attention to the use of antireflux procedures in the surgical treatment of pancreatic achalasia. And the choice of which modality to perform surgery in patients with previous surgical history is also one of the points to be discussed.

## 1. Introduction

Gastric cardia carcinoma is one of the most important cancers in the world, and its occurrence is related to family genetic history, gastroesophageal reflux disease (GERD), *Helicobacter pylori (H. Pylori*) infection, and a variety of genetic risk factors. There is no clear clinical evidence of a correlation between achalasia and gastric cardia carcinoma, but the achalasia is associated with esophageal adenocarcinoma. After surgical and medical treatment of achalasia, patients are often reported to have symptoms of acid reflux, which may evolve into GERD. GERD is one of the risk factors for gastric cardia cancer, and this article aims to remind surgery to pay attention to the application of anti-reflux surgery when treating achalasia.

## 2. Case report

The patient, female, 85 years old, height 160 cm, weight 60 kg, BMI 23.4, body surface area of 1.49 m^2^, was admitted to our hospital with swallowing discomfort for 6 months. The patient underwent a Heller myotomy with achalasia in 2006, and occasionally had symptoms of acid reflux and heartburn after surgery, aggravated after eating food, self-oral omeprazole relief. No history of hypertension, no history of diabetes, no history of drug use, no family disease similar to the patient, no family genetic predisposition disease. The patient was initially diagnosed with gastric cardia cancer.

**Figure 1.**
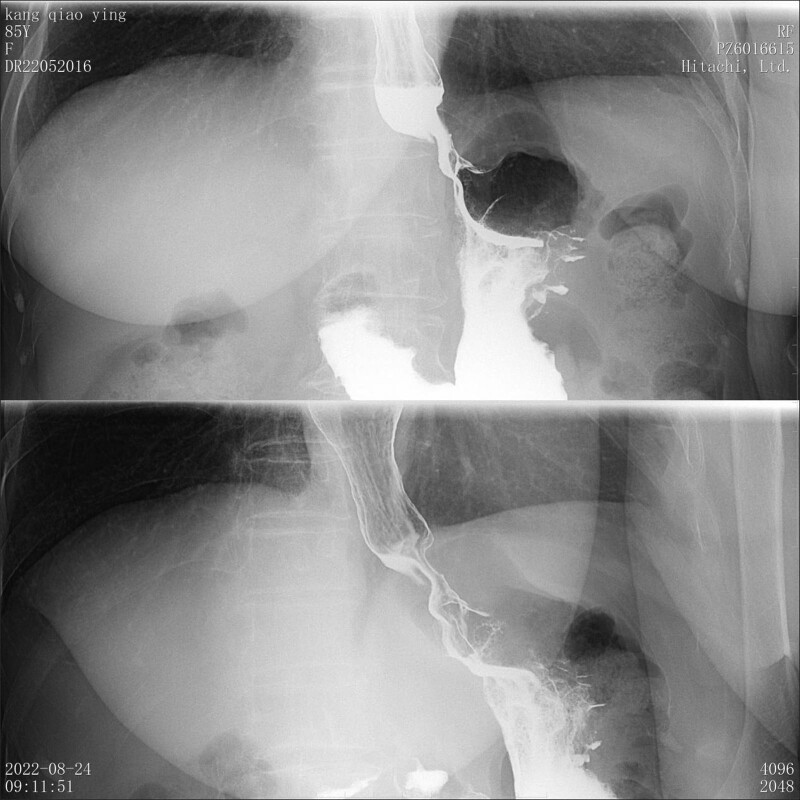
Result of preoperative upper gastroenterography the obstruction of barium through the cardia, stiff local tube wall, and irregular filling defect signs.

The clear consciousness, normal dietary intake and normal bowel movements which enter the hospital are suggestive of stable vital signs of the patient. Vital signs: body temperature: 36.5ºC, pulse: 108 beats/min, breathing: 19 times/min, blood pressure: 125/65 mm Hg. Superficial lymph nodes are not enlarged. Expansion symmetrical and normal, respiratory mobility is consistent, speech tremor is consistent, percussive percussion is clear and the breathing sounds are clear witch are in both lungs. Rhythm alignment. No pathological murmur, incision healing scar in the abdomen, soft abdomen, no obvious tenderness and rebound pain. No edema was observed in both lower limbs. Laboratory tests showed no abnormalities. Result of preoperative upper gastroenterography suggests gastric cardia cancer (Fig. 1). Preoperative pathologic analysis prompt middle—low differentiated adenocarcinoma.

## 3. Treatment

Improve the relevant examination and preoperative preparations, and enter the operating room on August 30, 2022 for radical resection of laparotomy gastric cardia carcinoma plus abdominal adhesion release. Establish an internal jugular vein channel, indulge the urinary catheter, and take a supine position after anesthesia is satisfied. Take the middle incision in the upper abdomen (the original incision), routinely disinfect the sheet, and use the incision as the center to lower the umbilical cord and up to the saber process. Incision into the abdominal cavity layer by layer, gradually separating the adherent abdominal cavity, detecting that the lesion is located in the gastric cardia door, excision of the omentum, preservation of the right blood vessel of the omentum, cutting off the left and short blood vessels of the stomach, and performing double ligation. Free gastric cardia, cut off the esophagus at a distance >3.0 cm from the tumor, remove 1/2 of the proximal segment of the gastric body, cut off the remaining gastric body on the excision line after closing with a cutting suture, remove the tumor, and at the same time sweep the lymph nodes in the drainage area. After anastomosing the end of the esophagus with the end of the posterior wall of the residual stomach with a stapler, the gastric tube is sent into the residual gastric cavity and the nutrient tube into the duodenum. The closure device closes the opening of the residual stomach, reinforces the gastric section and embedding, reinforces the anastomotic opening, tightly stops the bleeding, flushes the wound cavity, uses hemostatic material locally on the wound, and replaces 2 abdominal drainage tubes, which are led out from the upper abdomen. Determination of inactive bleeding spots on the wound, suturing the peritoneum, flushing of the incision, suturing of the white line, suturing of the incision layer by layer and bandaging. After the operation, the patient wakes up and returns safely to the ward. Postoperative topographic treatment of omeprazole sodium acid inhibition stomach protection, daheparin sodium anticoagulant, liquid supplementation and other symptomatic supportive treatment.

## 4. Results and the follow-up

**Figure 2.**
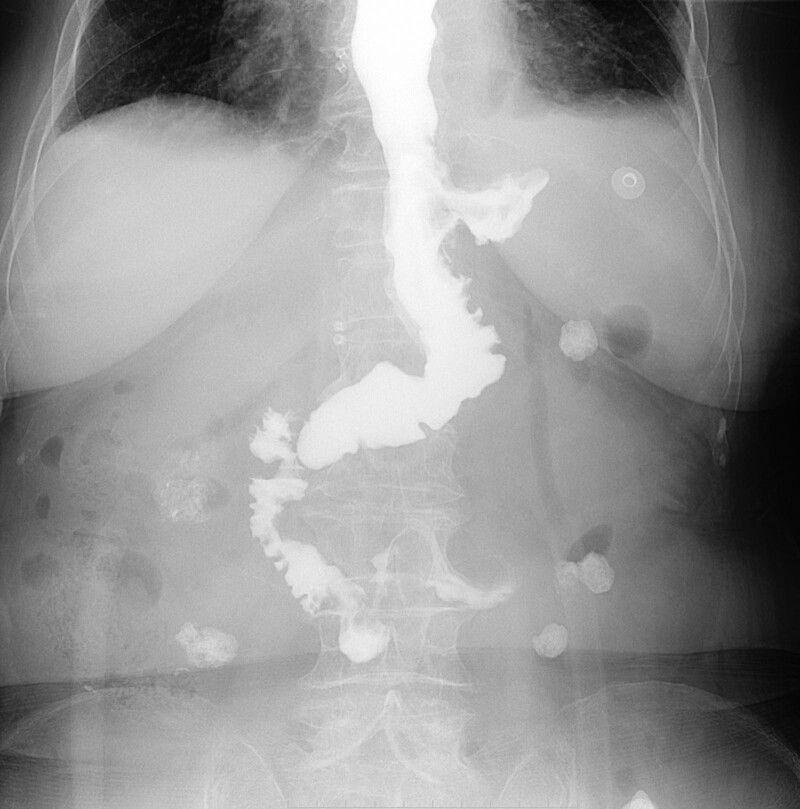
Result of Postoperative upper gastroenterography the anastomotic mouth is unobstructed, no spillover, residual stomach filling and duodenal scinning.

Result of cupper gastroenterography shows that the patient have shown no abnormal symptoms (like anastomotic leakage) so far (Fig. 2). The patient said that she felt the love of medical staff and the care of the surgeon during the hospital, and there were no adverse reactions after surgery. The patient was discharged with a better health condition. Postoperative pathological prompts that ulcerative low-medium differentiation adenocarcinoma in the junction of esophagus and stomach, Lauren classification is mixed type, subserous invasion, visible nerve invasion, no exact intravasculature carcinoma emboli, invasion of the lower esophageal segment. No cancer was found in the 2 resected margins of the examination and the omentum. Lymph nodes are seen with cancer metastases (small bend of the stomach 2/9). Pathological stage has shown Her-2(0), Ki-67 (80%+), MLH-1(+), PMS2(+), MSH2(+), MSH6(+), EGFR(+). According to immunohistochemical results suggest that tumor tissue mismatch repair protein expression intact (pMMR) and the histopathologic stage is pT3N1Mx.

## 5. Discussion

Gastric cancer is an important cancer worldwide, with more than 1 million new cases and an estimated 769,000 deaths reported in 2020 (equivalent to 1 in 13 deaths globally), ranking fifth globally in terms of incidence and fourth in terms of mortality. The incidence is twice as high in men as in women.^[1]^ Gastric cancer is classified according to anatomical site into gastric cardia adenocarcinoma (GCA) and noncardia gastric adenocarcinoma (NCGA). GCA originates in the region of the stomach adjacent to the oesophagogastric junction, whereas NCGA originates in the more distal region of the stomach, and the 2 subtypes differ in terms of etiology, risk factors, and geographic patterns. Similar to the etiology attributed to adenocarcinoma of the esophagus, GCA is associated with obesity and GERD, whereas the vast majority of NCGA cases are associated with *H Pylori* infection.^[2]^ Epidemiologically and biologically, GCA is thought to be distinct from adenocarcinomas located in the distal esophagus or stomach. In addition, GCA is usually at an advanced stage with a poor prognosis when detected. It may be associated with family history, demographic and behavioral factors, GERD, *H Pylori* infection, genetic risk factors, epigenetic risk factors, long stranded non-coding RNAs, MicroRNAs and other factors.^[3]^

The patient had a history of pancreatic achalasia, a disorder of esophageal motility with a reported^[4,5]^ global incidence and prevalence of 0.03 to 1.63 per 100,000 per year and 1.8 to 12.6 per 100,000 per year, respectively. Achalasia is a rare diagnosis in which is no racial preference for incidence in males and females, with peaks occurring predominantly between the ages of 30 to 60 years.^[4]^Patients typically present with progressive dysphagia, heartburn, chest pain, reflux, and varying degrees of weight loss or nutritional deficiencies.^[6]^After the diagnosis of achalasia, the patient underwent a cesarean Heller myotomy for treatment, and post-operative symptoms of acid reflux appeared, which worsened after eating, and oral omeprazole was administered to control the symptoms. In long-term follow-up, the majority of patients with pancreatic achalasia have symptoms of gastroesophageal reflux after surgical treatment, require dietary modifications and anti-reflux medications, and are receiving acid-suppressive therapy regardless of the type of surgery.^[7]^

There is a significant association between a single case of GERD and pancreatic cancer, which has been shown to increase the risk of pancreatic cancer by 2 to 4 times in patients with GERD in a number of studies.^[3]^Reflux symptoms are a risk factor for pancreatic cancer, and >2 GERD symptoms per week increases the risk of pancreatic cancer, but this correlation is only seen in the non-atrophic subtype of pancreatic cancer. And in relation to the histological subtype of cardia cancer, similar to esophageal adenocarcinoma, GERD symptoms are strongly associated with the intestinal subtype of cancer at the cardia, probably due to the fact that the intestinal subtype of cancer has some of the same mechanisms of occurrence as esophageal adenocarcinoma, e.g., gastric reflux leading to columnar intestinal metaplasia, xenografts, and adenocarcinoma. In contrast, there is no clear correlation between GERD symptoms and diffuse subtype adenocarcinoma at the cardia.^[8]^

What needs to be thought about in this case is that more attention needs to be paid to the prevention of reflux symptoms in the treatment of pancreatic achalasia. Currently, in the treatment of pancreatic achalasia with HM surgery or POEM surgery, it is possible to perform fundoplication, including Dor or Toupet fundoplication and Nissen procedure, which can be performed to achieve a reduction of postoperative reflux by a reasonable anti-reflux mechanism, but Dor or Toupet fundoplication is preferred to reduce dysphagia. Antireflux surgery can reduce the rate of postoperative heartburn by up to 80% and reduce the risk of oesophagitis and gastrointestinal strictures,^[9,10]^ thus further reducing the likelihood of cancer. In addition, the anatomical position of patients who have undergone surgical treatment will cause changes, and at this time, the choice of the type of surgical treatment becomes one of the main points of concern. In this case, the patient was directly treated with radical gastric cardia cancer surgery by dissection, although a better efficacy was achieved, at the age of 85 years old, laparotomy may not be the optimal solution.

In summary, cancers of the gastrointestinal tract pose a major public health challenge. Primary and secondary preventive measures remain the most important tools for the control of this group of malignant tumors, especially given their preventable nature and often dire prognosis.^[11]^Therefore, for cardia cancer, in addition to the common control of tobacco, alcohol and obesity,^[11]^ when reflux symptoms caused by various reasons occur, reasonable anti-reflux through surgical and non-surgical means can also play a role in reducing the risk of cancer, but this needs to be supported by further clinical evidence. The data presented in this article are based on written consent from the patients and their families.

## Author contributions

**Investigation:** Da-wei Wei.

**Writing – original draft:** Lin-qi Wen.
